# Kozeny-Carman theory for modeling of porous granular structures saturation with emulsion during imbibition process

**DOI:** 10.1371/journal.pone.0188376

**Published:** 2017-12-21

**Authors:** Olga Shtyka, Łukasz Przybysz, Mariola Błaszczyk, Jerzy Sęk

**Affiliations:** Department of Chemical Engineering, Lodz University of Technology, Lodz, Poland; Coastal Carolina University, UNITED STATES

## Abstract

The issue discussed in the current publication is a process of emulsions penetration in the granular media driven by the capillary force. The research work focuses on the study of rate and height of multiphase liquids penetration in a porous bed. Changes of the medium porosity and saturation level occurring as a result of pores obstruction by the droplets of an inner phase, were considered. The surfactant-stabilized emulsions with the different dispersed phase concentrations were investigated applying a classical wicking test. The modified version of Kozeny-Carman theory was proposed in order to describe the observed imbibition process in porous structures composed of spherical grains. This approach allowed to predict transport of emulsions considering an effect of bed saturation and porosity changes. In practice, the introduced concept can be appropriable in the numerous industries and scientific fields to predict the imbibition process of the multiphase liquids in granular structures regarding variation of the investigated bed permeability.

## Introduction

The porous media represent a vast majority of the known solid materials and are characterized by pore network structure, which can be typically filled with a liquid or gas. In practice, the porous media include the biological tissues (e.g. wood, hair, bones), soil and rock matrixes (i.e. zeolites, pumice, oil and gas reservoir), and artificial materials among which ceramics, cements, woven and non-woven synthetic filament, artificial skin [1–6].

The pore structure of media has an immense importance for the different fields such as mechanics, engineering, and fluid transport processes like infiltration, elution, percolation, and imbibition [1, 3, 7–10]. The imbibition is regarded as a spontaneous liquid movement in a porous medium driven by the capillary pressure and counterbalanced by the viscous drag force and the gravity acceleration [1, 11]. The displacement of a non-wetting phase or air by a wetting liquid occurs in void pores during the mentioned process [1].

The imbibition occurs as a result of the pressure difference and is referred in the literature as a result of the capillarity effect. It is observed in case when the adhesion force, between a penetrating liquid and pore surface, is stronger in comparison with the cohesion force between molecules in a permeant. The liquid imbibition in a porous bed depends on several factors: the properties of a permeant, i.e. viscosity and surface tension, liquid-pore surface interactions, and geometrical structure of the pore voids [1, 4, 11]. Thus, fluids transport through porous media driven by capillary force is regarded as the complicated issue, and therefore, has been of current and growing interest from side of industry and fundamental science.

In the literature, there are numerous mathematical models to characterize and predict the spontaneous imbibition occurring in the different porous media. Firstly, Washburn investigated the process of water imbibition and proposed the model for single-phase liquids, which is also known as Lucas-Washburn equation [2, 12]. Initially this model was applied for the process in a vertical tube, but further Lucas-Washburn equation was modified to extend its relevance and consequently, was applied for the complex porous media [13, 14]. Due to the fact that porous medium structure can extremely vary, the several geometrical parameters were considered and discussed: i) average pore radius [1, 15–17]; ii) tortuosity and shape of pores [8, 13, 15, 18–20]; and iii) roughness of pore wall [21–25]. The influence of grains roughness formed a porous bed on the contact angle and adhesion was investigated in the work of Marmur (2009) [26]. Additionally, the conducted experiments proved that roughness of beads surface induced a strong effect on the kinetics of spontaneous imbibition [22, 27]. The models described the spontaneous imbibition in complex porous structures on the basis of fractal geometry [13, 14]. The model proposed by Hammecker *et al*. (1993) is mainly used to predict the kinetics of imbibition occurring in the sedimentary rocks. The investigated porous medium consisted of spherical, conical, sinusoidal, and elliptical elements [28]. Another approach found in the literature, allowed to predict wicking process in the unconsolidated granular materials [29]. A liquid imbibition was also studied in the heterogeneous porous structures composed of the sections with variations in radius [5].

There is a group of the mathematical models, which considered an effect of dynamic contact angle on the capillary rise of a single-phase permeant [16, 30–32]. To apply such models, the porous media were represented as an assembly of vertical parallel capillaries. These approaches gave the possibility to define and characterize the spontaneous imbibition in a wide range of porous media with different morphology.

To conclude, a vast majority of proposed mathematical equations predict imbibition as changes of the height of liquids front in a porous bed or changes of imbibed liquid mass versus time. Moreover, these models are appropriable only in case of single-phase liquids penetration as well as the represented experimental data regarding only on water [11, 16, 17, 33, 34], and different organic substances as dodecane, decane, hexadecane, dimethyl silicone oil, vegetable oil and kerosene etc. [1, 30, 33, 35].

This research work focuses on the investigation of emulsions penetration in a granular bed driven by the capillary suction pressure. An emulsion as a heterogeneous multiphase system, is composed of at least two immiscible compounds with the divergent physicochemical properties, i.e. viscosity, density, and wettability. Therefore, a porous bed imbibition with an emulsion is assumed to differ from the same process with a single-phase liquid.

In the current publication, the experiments were conducted to study changes of the imbibed emulsion height versus time and the saturation of a porous bed with the dispersed phase versus penetration level. The saturation was defined as a ratio between a volume of pores filled with the dispersed phase and a total volume of voids. The influence of the initial inner phase concentration and surfactant fraction on the imbibition process was also an objective of the current discussion.

The previously mentioned models were applied to characterize the imbibition phenomenon in case of a single-phase permeant. On the other hand, the heterogeneity of a permeant composition and the effect of porous bed porosity variation were disregarded in the proposed approaches. In this work, the modified version of Kozeny-Carman theory [36] was introduced to describe two-phase liquids imbibition considering the changes of medium saturation. The knowledge of imbibition mechanisms and the ability to predict this process in granular media is practically important for a wide range of industries and branches of science, mainly: oil and its emulsions recovery from fractured reservoirs, penetration of water and antifungal emulsions into the different building materials (i.e. concrete, cement, etc.), studies of hydrological regime in soils and migration process of water and contaminants through the vadose zone.

## Materials and methods

### Materials

In the presented experiments, the investigated emulsions were composed of oil (EOL Polska Sp.z.o.o., Poland) as a dispersed phase and distilled water as a continuous phase. The dispersions differed by the inner phase concentration *φ*_*d*_, which was equal to 10 vol%, 30 vol%, and 50 vol%. The oil viscosity was 53.7 ±0.7 mPa·s and surface tension was 39.3 mN/m. The density was equal to 920 ±1.7 kg/m^3^ and API density was 22.63°. The investigated emulsions were stabilized by a surfactant with a fraction *φ*_*s*_ of 1 vol%, 2 vol%, and 5 vol%. Its viscosity was 50.2 ±0.6 mPa·s. The surfactant had density of 908 ±2.7 kg/m^3^ and surface tension of 36 ±1.8 mN/m. The emulsions were prepared by a mechanical stirring during 600 s (homogenizer MSM–67170 Bosch). The dispersed droplets in the investigated emulsion had the size of 1–20 μm, and 70–80% of them was in a range of 1–5 μm. The properties of the dispersions are shown in Table 1.

**Table 1:** Physicochemical characteristics of emulsions, T = 23 ±1°C.

Fraction of surfactant	Dispersed phase concentration		
	10 vol%	30 vol%	50 vol%
Density, kg/m^3^			
**1 vol%**	990.4±1.70	975.9±2.18	960.0±1.51
**2 vol%**	990.0±1.31	973.9±1.43	959.1±2.18
**5 vol%**	987.7±1.16	972.4±3.12	957.5±1.09
Viscosity, mPa·s			
**1 vol%**	5.61±0.11	11.23±0.33	27.54±0.93
**2 vol%**	5.92±0.14	14.85±0.23	29.80±0.24
**5 vol%**	10.18±0.21	33.98±0.53	69.38±0.83

The used porous bed was composed of 500–700 μm glass beads (Alumetal-Technik, Poland). The average diameter was equal to 620.1 ±8.2 μm. The investigated porous bed revealed oleophilic/ hydrophilic property. The porosity of this granular medium was 0.37 ±0.012, and the bulk density equaled 1731 ±2.1 kg/m^3^.

### Experimental procedure

The emulsions imbibition in granular media was investigated experimentally due to the classical wicking test. A column filled with the spherical grains (*d*_*m*_ of 0.035 m and *h*_*m*_ of 0.15 m) was immersed in a beaker with a dispersion. The cross-section of the used column was 9.61×10^−4^ m^2^. The imbibition process was followed till time *t*_*eq*_ when the mass change of an imbibed emulsion was smaller than 0.02 g per 200 s. The height observed at *t*_*eq*_ was considered as the maximal one and denoted as *h*_*eq*_. The changes of an emulsion front rise in a porous bed was also determined with time and noticed as *h*_*im*_. After a wicking test, a medium was removed from the beaker, and its soaked part was cut into fragments. Each fragment had identical dimensions, i.e. the height of 0.020 m. The concentration of an emulsion imbibed in the obtained fragments was measured using nephelometric method and microscopic analysis. This procedure was described in details in the earlier publications [10, 35].

### Analytical methods

The nephelometric method allows to define the dispersions concentration measuring the intensity of the scattered light by an optical analyser Turbiscan^TM^ LAB (Formulaction, France). The application of this method for the quantitative analysis of emulsions concentration was discussed in the publications of Lemarchand *et al*. (2003) and Shtyka *et al*. (2016) [10, 37]. In the current research work, the distribution of inner phase droplets in the obtained emulsions was measured by means of an analysis of their microscopic images (microscope Leica DMI3000B, camera Lumenera Infinity1).

The viscosity value was obtained using a shear rheometer Bohlin CVO-120 (Malvern Instruments, UK). Measurement of surface tension was performed by a ring pulling method, using a tensiometer KRÜSS K12 (KRÜSS GmbH, Germany). In order to ensure the repeatability and validity of the results, each measurement was performed three times. The experiments were conducted at the temperature of 23 ±1°C and atmospheric pressure.

## Results and discussion

### Experimental part

**Fig 1:**
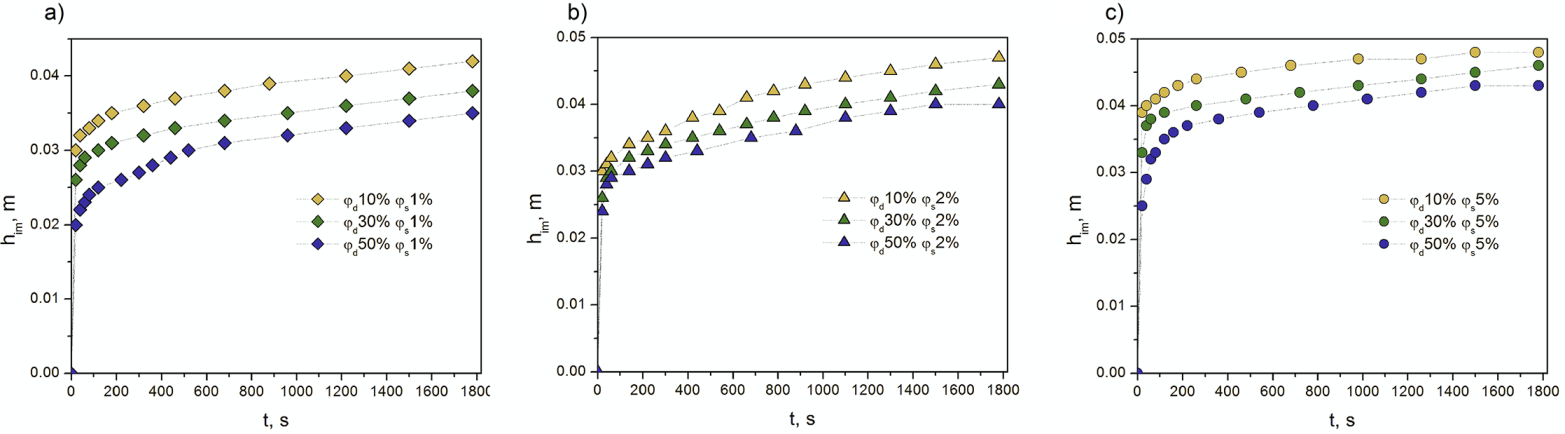
Changes of the height versus time for emulsions with the different surfactant concentrations. (A) surfactant concentration *φ*_*s*_ of 1 vol%, and dispersed phase concentration *φ*_*d*_ of 10–50 vol%. (B) surfactant concentration *φ*_*s*_ of 2 vol%, dispersed phase concentration *φ*_*d*_ of 10–50 vol%. (C) surfactant concentration *φ*_*s*_ of 5 vol%, dispersed phase concentration *φ*_*d*_ of 10–50 vol%.

The height of an emulsion penetration in a granular medium is considered as an important parameter to characterize the kinetics of imbibition process. The experimental results on the changes of a front height versus time are represented in Fig 1 http://link.springer.com/article/10.1007/s13762-016-1076-2-Tab2.

The emulsions with the dispersed phase of 10 vol% achieved the maximal height in all investigated cases. This value varied and depended on the added emulsifier concentration: *h*_*eq*_ was equal to 0.048 m for 5 vol%, 0.047 m and 0.042 m for 2 vol% and 1 vol%, respectively. Conversely, the lowest levels of penetration were observed in case of 50% emulsions, i.e. 0.043–0.035 m (Fig 1). Thus, the increase of a surfactant concentration caused rise of the penetration height in a granular medium. For 10% emulsions, the height was equal to 0.048 m for an emulsifier fraction of 5 vol%, and reduced by 7% for *φ*_*s*_ of 1 vol%. In case of 50% emulsions, it decreased by 18.6%, that is from 0.043 m (*φ*_*s*_ = 5 vol%) to 0.035 m (*φ*_*s*_ = 1 vol%). The same tendencies were observed for emulsions with the dispersed phase concentration of 30 vol% (Fig 1). The maximal value of experimental data error was equal to 6.3%.

**Fig 2:**
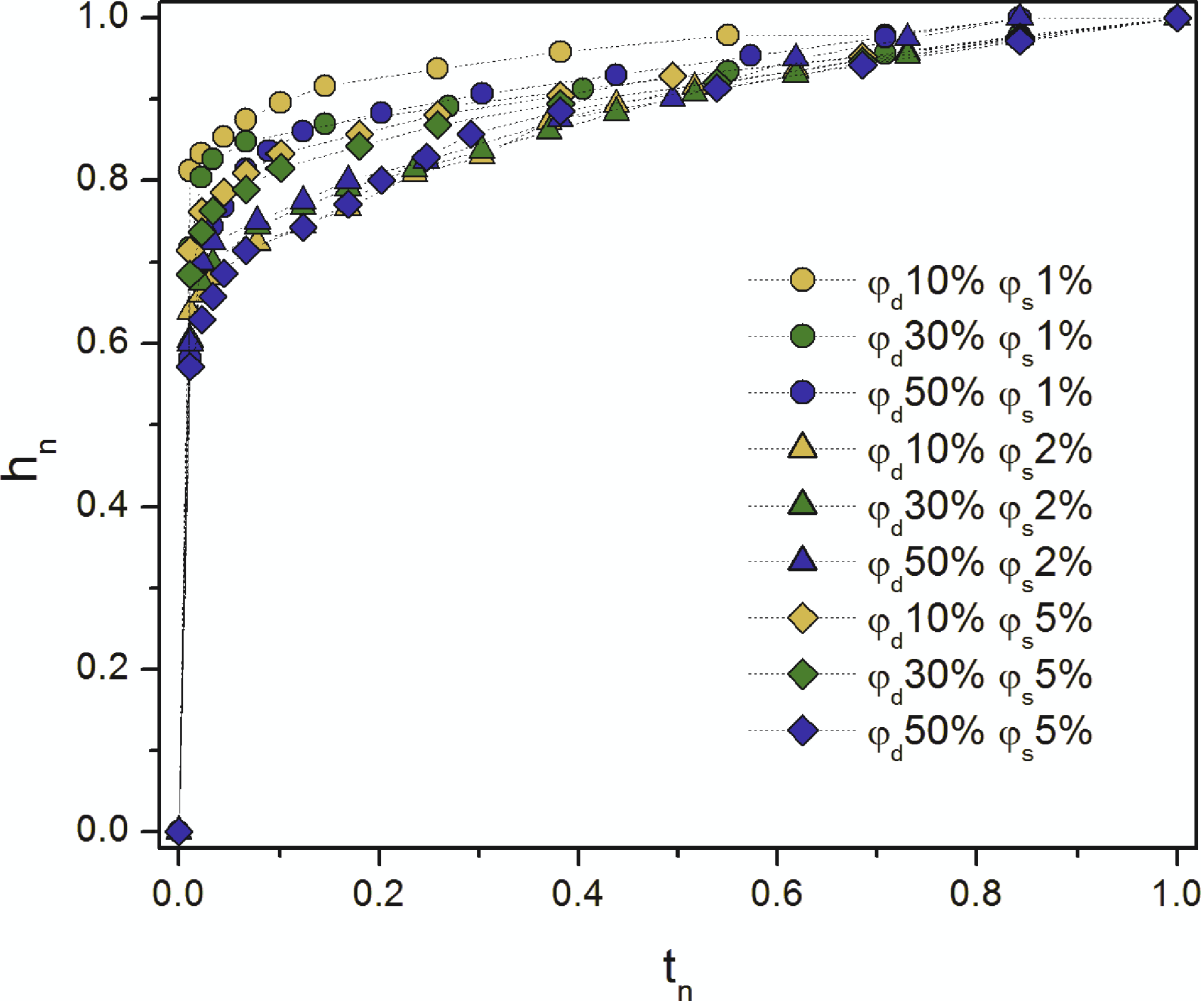
Comparison of the normalized height *h*_*n*_ changes versus normalized time *t*_*n*_.

On the graphs in Fig 2, the vertical axis represents the normalized values of the height of an emulsion front, which were calculated as a ratio of *h*_*im*_*/h*_*eq*,_ and the horizontal one shows the dimensionless time of imbibition process, which was defined as *t*_*im*_*/t*_*eq*_.

The results plotted in Fig 2 gave the possibility to summarize that the tendency of the imbibition process slightly differed in a normalized time interval of 0.05–0.6, and after it had almost the same trend for all investigated dispersion. Thus, a composition of the multiphase liquids has a significant influence on the height of their penetration in the granular media. Such phenomenon is observed due to the increase of surfactant and dispersed phase concentrations and consequently, changes of the viscosity. On the other hand, it is possible to assume that droplets were retained in lower layers of the investigated granular bed causing obstruction of the penetration paths.

**Fig 3:**
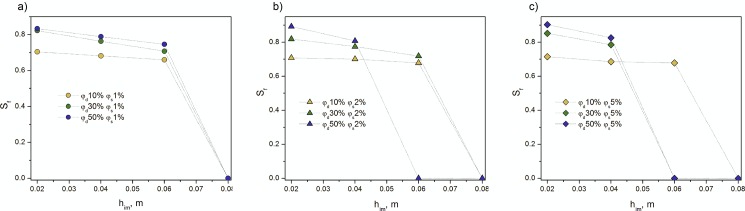
Changes of saturation with the dispersed phase versus the height of an imbibed emulsion front at the maximal time. (A) surfactant concentration *φ*_*s*_ of 1 vol% and dispersed phase concentration *φ*_*d*_ of 10–50 vol%. (B) surfactant concentration *φ*_*s*_ of 2 vol% and *φ*_*d*_ of 10–50 vol%. (C) surfactant concentration *φ*_*s*_ of 5 vol% and *φ*_*d*_ of 10–50 vol%.

The results on the changes of granular bed saturation with the dispersed phase versus the height at the equilibrium time *t*_*eq*_ are shown in Fig 3.

The saturation level strongly depended on composition of the two-phase liquids and an amount of the added surfactant. Thus, the higher values of the saturation were observed for 50% emulsions with *φ*_*s*_ of 1 vol% (Fig 3A), and up to 0.06 m in case of its fraction equaled 2 vol% and 5 vol% (Fig 3B and 3C). Consequently, the lowest saturation was obtained for emulsions with the dispersed phase of 10 vol% (Fig 3). The exception was emulsions with emulsifier fraction of 5 vol% for *h*_*im*_ = 0.06 m, because neither 30% nor 50% emulsions penetrated there (Fig 3C). In addition, the increase of an emulsifier caused slight enlargement of porous bed saturation with the dispersed phase (Fig 3). The saturation level tended to decrease with the increase of an emulsion penetration in the investigated granular medium (Fig 3).

To conclude, the highest level of emulsion penetration in a porous bed was observed in case of 10% emulsions. In contrast, the highest saturation in a majority of cases was observed for 50% emulsions. Moreover, the decrease of emulsifier concentration caused the reduction of the height of an imbibed emulsion in the medium as well as the changes http://www.lingvo.ua/uk/Search/Translate/GlossaryItemExtraInfo?text=%d0%bf%d1%80%d0%b8%d0%b1%d0%b0%d0%b2%d0%bb%d0%b5%d0%bd%d0%b8%d0%b5&translation=raise&srcLang=ru&destLang=en of bed saturation with the dispersed phase.

### Theoretical part

The Kozeny-Carman theory was used to describe the process of emulsions imbibition in granular medium. According to this approach, each granular medium can be regarded as a bundle of the tortuous capillaries through which a liquid penetrates:

fr=ARef, (1)

where *f*_*r*_ is the flow resistance coefficient, *Re*_*f*_ is the Reynolds number, and *A* is an equation coefficient [38]. The Reynolds number *Re*_*f*_ can be represented according to the following expression:

$$Re_{f}=\frac{d_{b}v\rho }{\eta (1-\varepsilon )},$$ (2)

where *η* is the viscosity of a liquid, *d*_*b*_ is the average diameter of glass beads in a porous bed, *ε* is the porosity of a granular bed, *v* is the velocity of a liquid flow, and *ρ* is the density of a penetrating liquid.

For the discussed case and considering that this process occurs under the isothermal conditions, the flow resistance coefficient can be expressed as:

$$f_{r}=\frac{\Delta p}{\nu^{2}\rho }\frac{d_{b}}{h_{w}}\frac{\varepsilon^{3}}{1-\varepsilon },$$ (3)

where *h*_*w*_ is the height of a wetted bed, and *Δp* is the change of pressure in the investigated porous structure.

The Kozeny–Carman theory was originally developed to predict flow of a single-phase liquid. In the current publication, this model was applied to describe transport of an emulsion as two-phase liquid during imbibition process. Firstly, the porosity of a granular bed was replaced by the relative porosity *ε*_*r*_. This parameter is frequently represented as the difference between a volume of pores and volume occupied by the dispersed phase, divided by a total volume of such porous medium. In this case, the relative porosity is expressed considering the saturation level of a granular structure:

$$\varepsilon_{r}=\varepsilon (1-S_{f}),$$ (4)

where *S*_*f*_ is the degree of bed saturation with the dispersed phase.

To consider the initial concentration of the dispersed phase *φ*_*d*_ in an emulsion, the special coefficient *k*_*d*_ was applied, which is expressed as the dependence:

$$k_{d}=\delta \varphi_{d},$$ (5)

where *δ* is a coefficient of Eq (5), which is equal to 0.1 for all investigated emulsions in this case.

After substitution of Eq (4) in Eq (3) and considering Eq (5), one can obtain:

$$f_{{}_{r}}^{*}=k_{d}\frac{\Delta p}{\nu^{2}\rho }\frac{d_{b}}{h_{w}}\frac{{\left(\varepsilon (1-S_{f})\right)}^{3}}{1-\varepsilon (1-S_{f})}.$$ (6)

The velocity of liquid penetration can be defined according to:

$$v=\frac{\Delta h}{\Delta t},$$ (7)

and *Δp* is calculated using the following equation:

$$\Delta p=p_{c}-p_{h},$$ (8)

where *p*_*c*_ is the capillary pressure, and *p*_*h*_ is the hydrostatic pressure [2, 3, 11].

The capillary pressure is calculated as:

$$p_{c}=\frac{2\sigma \cos\theta }{r_{h}},$$ (9)

where *σ* is the surface tension, *θ* is the contact angle, and *r*_*h*_ is the hydraulic radius, which can be obtained according to the modified Kozeny–Carman approach:

$$r_{h}=\frac{1}{6}d_{b}\frac{\varepsilon_{r}}{1-\varepsilon_{r}}.$$ (10)

The hydrostatic pressure is defined due to the following expression:

$$p_{h}=\rho g\Delta h,$$ (11)

where *g* is the gravity force. To substitute Eqs (9), (10) and (11) in Eq (8), one can obtain:

$$\Delta p=\frac{12(1-\varepsilon (1-S_{f}))\sigma \cos\theta }{d_{b}\varepsilon (1-S_{f})}-\rho g\Delta h.$$ (12)

In the discussed case, when a penetrating liquid is assumed to be completely wetting, thus *cosθ* = 1. After simplification and arrangements, the final equation to determine the flow resistance coefficient can be represented as:

$$f_{{}_{r}}^{*}=k_{d}(\frac{12(1-\varepsilon (1-S_{f}))\sigma }{d_{b}\varepsilon (1-S_{f})}-\rho g\Delta h)\frac{d_{b}}{\nu^{2}\rho h_{w}}\frac{{\left(\varepsilon (1-S_{f})\right)}^{3}}{1-\varepsilon (1-S_{f})}.$$ (13)

In Eq (2), the porosity of a granular bed can be also displaced by the relative porosity, and therefore Eq (4) is substituted into Eq (2). Finally, the Reynolds number may be calculated as:

$$Re_{f}^{*}=\frac{d_{b}v\rho }{\eta (1-\varepsilon (1-S_{f}))}.$$ (14)

**Fig 4:**
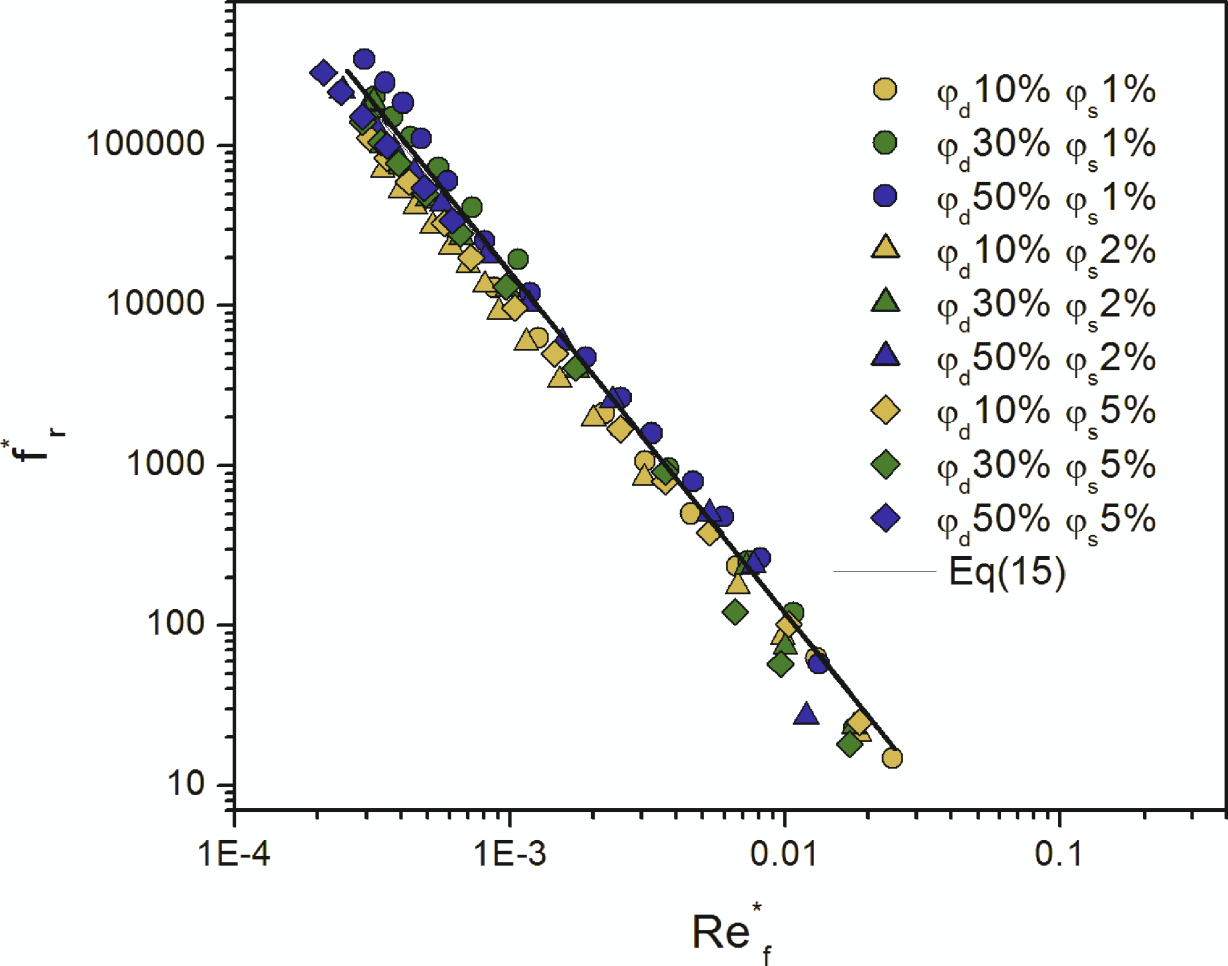
Dependence between the flow resistance coefficient $f_{{}_{r}}^{*}$ and the Reynolds number $Re_{f}^{*}$ for the investigated emulsions.

Thus, the Reynolds number $Re_{f}^{*}$ and the flow resistance coefficient $f_{{}_{r}}^{*}$ were calculated due to Eqs (13) and (14), respectively, and their relation was presented on the graph in Fig 4.

As shown in Fig 3, the obtained dependence may be described using the following equation:

fr*=α(Ref*)β, (15)

where *α* and *β* are the parameters of the proposed model and are equal to 0.02±0.0017 and -2.0±0.04, respectively.

Thus, the proposed concept allows to predict the porous granular medium saturation with the oily compositional phase during process of the multiphase liquids imbibition. To compare with other approaches, it provides the possibility to define the variation of the porosity with the height of a permeant penetration driven by the capillary force and considers the structural parameters of a porous medium. It can be relevant in the fundamental science and is of importance in industrial sector due to the fact that many processes are based on the capillary rise phenomenon.

## Conclusions

To conclude, the height of emulsion wicking in a porous granular medium depended on emulsifier and dispersed phase concentrations. The saturation level decreased with the height of emulsions penetration in the porous structure and the composition of a permeant, i.e. fraction of the added surfactant and inner phase concentration influenced strongly on its changes.

The modified version of Kozeny–Carman theory was used to describe the observed imbibition process in the porous medium composed of spherical grains. This approach allowed to predict transport of the emulsions considering effect of bed saturation and porosity changes.

The introduced concept is applicable in the numerous industries to predict the imbibition process of the multiphase liquids in the granular structures regarding variation of the bed permeability.
